# Application of Gene Editing Technology in Poultry

**DOI:** 10.3390/vetsci13050484

**Published:** 2026-05-17

**Authors:** Ruyu Liao, Rong Ran, Yixin Liu, Xinyi Zhou, Min Tan, Qigui Wang, Haiwei Wang, Xi Lan

**Affiliations:** 1 College of Animal Science and Technology, Southwest University, Chongqing 400715, China; a3029561195@email.swu.edu.cn (R.L.); wrjrk154927@email.swu.edu.cn (R.R.); cq18696963882@email.swu.edu.cn (Y.L.); a524448729@email.swu.edu.cn (X.Z.); tanmin123@email.swu.edu.cn (M.T.); 2 Chongqing Academy of Animal Sciences, Chongqing 402460, China; wnagqigui@hotmail.com

**Keywords:** gene editing, CRISPR/Cas9, delivery vector, animal breeding, poultry animals

## Abstract

Gene editing technology, a revolutionary tool in molecular biology, enables precise modifications of genomic sequences and gene expression patterns, thereby conferring desired traits to cells or organisms. Since 2014, CRISPR/Cas9 has rapidly become the most widely used gene editing method in agricultural animals due to its high editing efficiency. Subsequently, the development of novel gene editing systems, such as base editors and prime editors, has provided enhanced precision and reduced off-target effects. These advancements have facilitated the transition of gene editing from laboratory research to clinical and agricultural applications. Gene editing has been extensively utilized to enhance production traits, improve disease resistance, facilitate disease detection, and establish disease models. This review outlines the development of gene editing technologies, discusses the advantages and limitations of key gene editing tools, and explores their applications in poultry. Furthermore, it examines the challenges and future prospects of gene editing in animal husbandry, including off-target effects, ethical concerns, and technical complexities.

## Simple Summary

Gene editing has become a key technology in poultry research. Common methods include ZFNs, TALENs, CRISPR/Cas9 and others, among which CRISPR/Cas9 is the most widely used due to its simplicity and high efficiency. This paper reviews several common gene-editing techniques and their applications in enhancing disease resistance, improving production performance, and detecting diseases. Finally, the existing challenges and future opportunities are summarized.

## 1. Introduction

**Figure 1:**
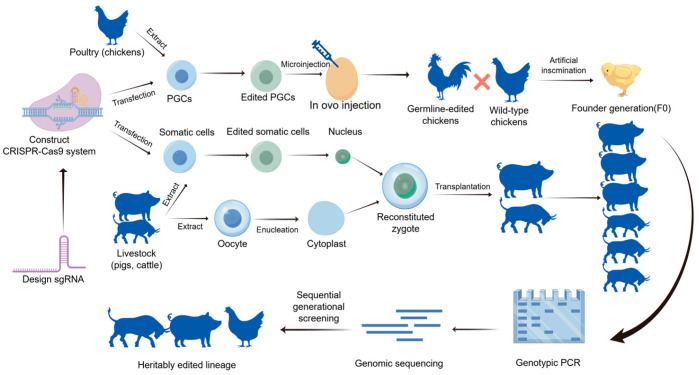
The CRISPR/Cas9 gene editing process for livestock and poultry. The schematic illustrates the systematic workflow for generating gene-edited animals. Initially, specific sgRNAs are designed to construct the CRISPR/Cas9 expression vectors. For poultry (e.g., chickens), primordial germ cells (PGCs) are extracted, transfected in vitro, and then injected into developing embryos to generate chimeric founders (F0), which are subsequently crossed to produce heritable germline-edited lineages. For livestock (e.g., pigs, cattle), somatic cells are edited and utilized as donor cells for somatic cell nuclear transfer (SCNT). The reconstituted zygotes are transplanted into surrogate mothers. Finally, the heritably edited lineages are validated through genotypic PCR, genomic sequencing, and sequential generational screening to ensure the stability and accuracy of the targeted traits.

Gene editing technology utilizes molecular biology techniques to precisely alter genomic sequences and gene expression patterns, enabling cells or organisms to acquire desired traits. In recent years, gene editing has advanced rapidly, with traditional gene editing technologies, including the first-generation ZFN technology, the second-generation TALEN technology, and the third-generation CRISPR/Cas9 technology [1]. Since 2014, various Cas9 variants, as well as base editors and prime editing, have been developed [2]. With continuous technological advancements, gene editing has progressively expanded from laboratory research to clinical applications and has been extensively studied for modifying biological traits in agricultural animals, demonstrating significant potential for novel breed development and enhancement of livestock production efficiency. Compared to conventional breeding methods and genomics-assisted breeding approaches, gene editing-based breeding offers distinct advantages. For instance, while traditional breeding is limited to selecting existing traits within a population, gene editing enables the precise introduction of novel target traits. Moreover, this technology significantly accelerates the breeding process and circumvents the selection of undesirable traits caused by linkage disequilibrium in conventional breeding programs. Taking the CRISPR/Cas9 gene editing system as an example (Figure 1), genetic modification in agricultural animals begins with the identification of candidate genes based on research objectives, followed by the design of target-specific gRNAs using bioinformatics tools. Subsequently, molecular cloning techniques are employed to integrate the gRNA and Cas9 nuclease sequences into expression vectors, incorporating selectable markers to facilitate subsequent identification. Upon completion of vector construction, an appropriate delivery method must be selected.

In livestock, plasmid delivery is typically achieved through somatic cell nuclear transfer (SCNT) or pronuclear microinjection of zygotes, with the edited positive embryos cultured in vitro to the blastocyst stage before being transferred into synchronized recipient females for gestation completion. In poultry, primordial germ cells (PGCs) serve as the primary editing vehicle, where in vitro-edited PGCs are transplanted into avian embryos to produce chimeric offspring. Postnatal individuals must undergo validation of target gene editing efficiency via PCR and sequencing techniques, complemented by whole-genome sequencing to assess potential off-target effects. To ensure stable inheritance of the edited traits, homozygous individuals are obtained through successive generations, ultimately establishing genetically edited breeds or lines with the desired phenotypes. Currently, gene editing technology has been applied to improve production traits, enhance disease resistance, and disease detection. This paper will review the development of gene editing technology, discuss the advantages and disadvantages of several gene editing techniques, and examine the application of gene editing in poultry animals, along with the challenges it faces in this field.

## 2. Development of Gene Editing Technology

**Figure 2:**
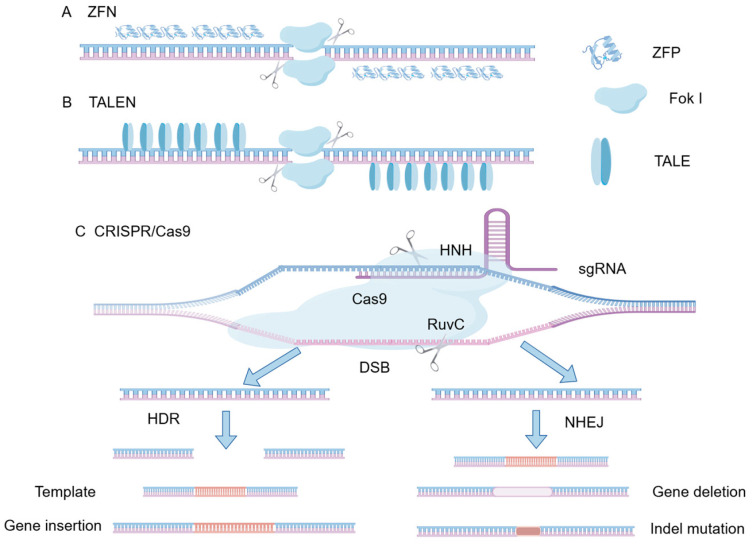
Schematic diagram of the structure of ZFN, TALEN and CRISPR/Cas9. (**A**) Zinc Finger Nucleases (ZFNs) consist of customizable zinc-finger DNA-binding proteins fused with a FokI endonuclease cleavage domain. (**B**) Transcription Activator-Like Effector Nucleases (TALENs) utilize TALE repeat arrays for DNA recognition, similarly tethered to a FokI nuclease. (**C**) The CRISPR/Cas9 system employs a single guide RNA (sgRNA) to direct the Cas9 nuclease to a specific genomic locus adjacent to a Protospacer Adjacent Motif (PAM). The Cas9 enzyme utilizes its HNH and RuvC domains to introduce a double-strand break (DSB). The resulting DSBs are subsequently repaired either by Non-Homologous End Joining (NHEJ), typically leading to gene deletion or indel mutations, or by Homology-Directed Repair (HDR) in the presence of a repair template, enabling precise gene insertion or modification.

**Figure 3:**
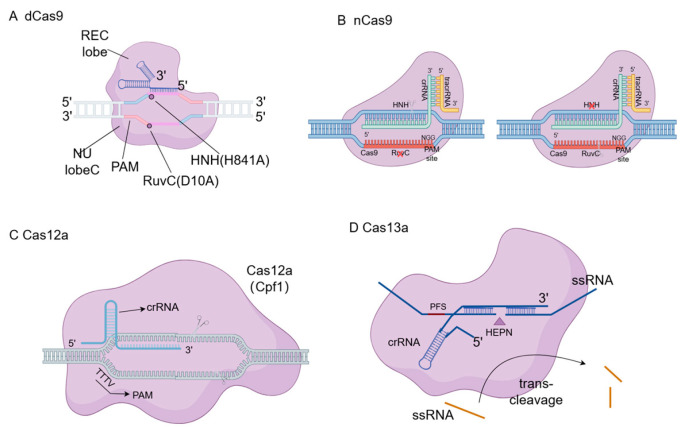
Structural diagrams of dCas9, nCas9, Cas12a and Cas13a. (**A**) Dead Cas9 (dCas9) contains inactivating mutations in both the RuvC and HNH nuclease domains, allowing it to bind to target DNA without causing cleavage, which is utilized for gene regulation (CRISPRa/CRISPRi). (**B**) Nickase Cas9 (nCas9) possesses a mutation in one of the nuclease domains, generating single-stranded DNA nicks rather than double-strand breaks, thereby reducing off-target effects when paired with dual sgRNAs. (**C**) Cas12a (Cpf1) relies on a single crRNA and a T-rich PAM sequence to create staggered double-strand breaks distal to the PAM. (**D**) Cas13a specifically targets and cleaves single-stranded RNA (ssRNA), possessing a unique collateral trans-cleavage activity upon target recognition, which is widely utilized in diagnostic biosensors.

Gene editing technology essentially involves the use of specially designed nucleases to create double-strand breaks (DSBs) at specific locations (S) in the genome of an organism. The cell’s own repair mechanisms, including non-homologous end joining (NHEJ) and homologous recombination (HDR), then repair the damaged genome, allowing for specific modifications such as insertions, deletions, or replacements at the target gene [3]. Traditional gene editing technologies include zinc finger nucleases (ZFNs), transcription activator-like effector nucleases (TALENs), and RNA-guided DNA endonucleases based on clustered regularly interspaced short palindromic repeats (CRISPR) and CRISPR-associated protein 9 (Cas9) (Figure 2) [4]. CRISPR/Cas9 technology has demonstrated significant advantages in terms of time and cost, with gene editing efficiency generally surpassing that of ZFNs and TALENs, making it the primary gene editing method in current agricultural animal research. Since 2014, it has been widely applied in livestock and poultry cell lines and species. Derived from CRISPR/Cas9, gene editing tools such as dCas9, nCas9, Cas12a (Figure 3), and novel gene editing technologies like base editors and prime editing have shown great potential in future animal husbandry applications due to their high targeting efficiency and low off-target activity (Figure 3). Gene editing technology has evolved over time, with several revolutionary breakthroughs since the 1970s, becoming an essential tool in life science research and medical applications. The development timeline is illustrated in Table 1, with key technologies introduced.

### 2.1. CRISPR/Cas9

The limitations of the first two generations of gene editing technologies drove the development of the third generation, CRISPR/Cas9. CRISPR is a sequence of short palindromic repeats found in prokaryotic genomes, first discovered in *E. coli* in 1987 [21]. The CRISPR sequence has three parts: a leader region, conserved repeats, and variable spacers. Cas refers to CRISPR-associated proteins. The Cas9 nuclease complex consists of the Cas9 protein, a specific CRISPR RNA (crRNA), and a trans-activating CRISPR RNA (tracrRNA). CRISPR/Cas9 serves as a defense mechanism, allowing bacteria to resist viral infections and phage invasions [22]. The antimicrobial defense mechanism of the bacterial CRISPR/Cas9 system against exogenous pathogens comprises three distinct phases. When foreign DNA first invades the host, the Cas1 and Cas2 endonucleases recognize and cleave the viral genome, inserting fragments into the leader region of the bacterial CRISPR sequence while replicating the existing repeat sequences to form new repeats. When foreign DNA invades again, the leader region initiates the transcription of the CRISPR sequence into a precursor crRNA (pre-crRNA), which is then processed by the Cas9 protein to form crRNA. The crRNA and tracrRNA form a dimer and bind to the Cas9 protein, forming a crRNA-Cas9-tracrRNA complex. This complex is then degraded by RNase III, producing small crRNA guides that target and cleave the foreign DNA [1,23,24].

The artificially designed CRISPR/Cas9 system consists of a duplex RNA (tracrRNA/crRNA) and the Cas9 endonuclease bound to the RNA strand. The crRNA region is approximately 20 nucleotides long and contains a guide RNA (gRNA) that binds to the target DNA sequence. The tracrRNA consists of a 14-nucleotide anti-repeat region and three loops. The targeting specificity of the CRISPR/Cas9 system is partly determined by the base pairing between the gRNA and the target DNA, and partly by the Cas9 protein and a short DNA sequence known as the protospacer adjacent motif (PAM), typically composed of the NGG sequence, where N can be any of the four DNA nucleotides recognized by the Cas9 nuclease. The PAM sequence is located at the 3′ end of the target DNA. Before designing the gRNA, a suitable PAM sequence must be identified in the genome. Cas9 initiates the target site search process by detecting the preferred PAM sequence, allowing the gRNA to precisely bind to the target DNA. Crucially, the DNA targeting and hybridization mechanism of the CRISPR/Cas9 system exhibits distinct structural and functional polarity. The recognition process is highly directional; following PAM recognition at the 3′ end of the target DNA strand, the unwinding of the DNA double helix and the subsequent RNA-DNA heteroduplex formation (R-loop) propagate in a polar, zipper-like manner. This propagation proceeds strictly from the PAM-proximal “seed” region (typically the 8–12 nucleotides adjacent to the PAM) towards the PAM-distal 5′ end. This functional polarity fundamentally dictates the mismatch tolerance of the system: the PAM-proximal seed region is highly stringent and sensitive to base mismatches, whereas the PAM-distal end exhibits significantly greater flexibility. Understanding this directional polarity is essential for optimizing gRNA design to maximize on-target cleavage efficiency while mitigating off-target hybridization. The Cas9 helicase domain unwinds the double strand, while the nuclease domains (RuvC and HNH) execute the DSB in the DNA.

The CRISPR/Cas9 system was first proposed as an effective gene editing tool in 2012 and won the Nobel Prize in Chemistry in 2020 [25]. The CRISPR/Cas9 system is classified into types I, II, and III based on the characteristics of the Cas9 protein, with the type II prokaryotic CRISPR adaptive immune system proven to facilitate RNA-guided site-specific DNA cleavage. In 2013, Zhang et al. first used the type II CRISPR/Cas9 system to perform gene editing in mammalian cells [26]. The CRISPR/Cas9 system can recognize and cleave specific target DNA, creating double-strand breaks that induce the cell’s DNA repair mechanisms. In the absence of homologous DNA, repair occurs via NHEJ, leading to gene deletions [27]. When homologous DNA is present, repair occurs via HR, and by designing a single-stranded oligonucleotide (ssODN, or donor sequence), specific gene insertions can be achieved. Compared to ZFN and TALEN technologies, CRISPR is simpler, faster, and offers significant time and cost advantages. It also allows for simultaneous editing of multiple genes within a single cell, demonstrating high gene editing efficiency.

#### 2.1.1. Nickase Cas9 (nCas9)

Nickase Cas9 (nCas9) is generated by activating one of the nuclear domains (RuvC or HNH) of Cas9, resulting in a single-strand nicking enzyme [28]. Compared to traditional Cas9, nCas9 only cleaves one strand of DNA, significantly reducing off-target effects and providing higher editing precision. By designing two adjacent sgRNAs, nCas9 can induce a “double-nicking” strategy, where double-strand breaks are only triggered when both nicks are present, reducing off-target rates by 50 to 1500 times [29]. The two gRNAs form cuts at the first and fifth bases after the recognition site [30].

#### 2.1.2. dCas9

Dead Cas9 (dCas9) is a variant of Cas9 with deactivated nuclease activity through point mutations (e.g., D10A and H840A), retaining DNA-targeting capability but no longer cleaving DNA. This characteristic makes it an ideal tool for gene regulation. dCas9 can be fused with regulatory factors to activate or silence genes and modulate protein activity levels. For example, fusing dCas9 with transcriptional activators (e.g., VP64) or repressors (e.g., KRAB domain) enables gene activation (CRISPRa) or interference (CRISPRi) [31].

#### 2.1.3. CRISPR/Cas12a

The CRISPR/Cas12a system, like CRISPR/Cas9, belongs to the type II CRISPR system but has significant differences, offering an alternative for gene editing. Unlike Cas9, Cas12a’s activity is primarily mediated by a short crRNA. In the Cas12a system, the specificity of target DNA recognition is guided by a longer spacer sequence of at least 22–23 nucleotides, enhancing recognition specificity. Cas12a recognizes PAM sequences rich in thymine (T) rather than the guanine (G)-rich PAMs required by Cas9, expanding the range of potential target sites. Cas12a generates staggered DSBs distal to the PAM, whereas Cas9 produces DSBs proximal to the PAM. Cas12a cleaves the target DNA strand 23 nucleotides downstream of the PAM region. Similar to Cas9, only some Cas12a homologs exhibit significant activity in eukaryotic cells. Cas12a, like Cas9, has a bilobed structure, including REC and NUC lobes. The Cas12a system uses the RuvCnuclease domain and a crRNA with RNase WED III to cleave both DNA strands, while Cas9 uses two domains for cleavage. In type II-A systems, Cas9 recognizes the PAM sequence located directly downstream of the protospacer, requiring tracrRNA, whereas Cas12a only requires crRNA to cleave the target strand [32,33].

#### 2.1.4. CRISPR/Cas13

The CRISPR/Cas13 system is an RNA-targeting CRISPR system initially discovered in bacteria as a defense mechanism against RNA viruses. The CRISPR/Cas13 system achieves gene regulation by recognizing and cleaving single-stranded RNA (ssRNA) [34]. Upon binding to the target RNA, the Cas13 protein activates its nuclease activity, cleaving the target RNA and inhibiting its expression. It has been used to develop highly sensitive RNA detection tools.

### 2.2. Base Editor

**Figure 4:**
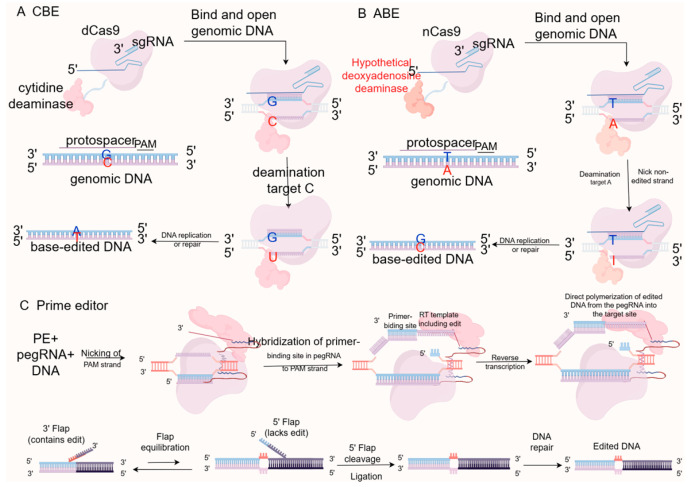
Operating principle of CBE, ABE and PE. (**A**) Cytosine Base Editors (CBEs) fuse a cytidine deaminase with dCas9 or nCas9 to catalyze the conversion of cytosine (C) to uracil (U), ultimately resulting in a C-G to T-A transition during DNA repair. (**B**) Adenine Base Editors (ABEs) employ an evolved deoxyadenosine deaminase to convert adenine (A) to inosine (I), which is treated as guanine (G) by the polymerase, leading to an A-T to G-C transition. (**C**) Prime Editors (PEs) consist of an nCas9 fused to a reverse transcriptase (RT) and utilize a prime editing guide RNA (pegRNA). The pegRNA not only specifies the target site but also provides the RNA template containing the desired edits (insertions, deletions, or substitutions), enabling highly precise genome modifications without requiring donor DNA templates or inducing double-strand breaks.

Traditional gene editing technologies require the creation of DNA double-strand breaks, leading to unavoidable biological damage. Both DSB repair pathways, NHEJ and HDR, have limitations. In most somatic cells, NHEJ repair is more efficient than HDR but is prone to erroneous base insertions or deletions. In contrast, HDR can precisely introduce desired changes, including insertions, deletions, or replacements based on a DNA repair template [35]. However, HDR is limited to the S/G2 phase of the cell cycle and is inefficient in most therapeutically relevant cell types [35,36]. The development of single-base editing technology based on CRISPR/Cas9 has achieved new breakthroughs in gene editing tools. Currently developed single-base editing systems include cytosine base editors (CBEs), adenine base editors (ABEs), guanine base editors (GBEs), and prime editors (PEs) (Figure 4). These systems enable the replacement of different bases, such as A-to-G, C-to-T, C-to-G, and C-to-A. The avoidance of DSBs significantly reduces the occurrence of unintended mutations, such as base insertions, deletions, or frameshifts, making these systems highly efficient and precise. They hold great significance in the establishment of disease models and gene therapy.

### 2.3. Prime Editing

Prime editing (PE) systems achieve gene editing at target sites by fusing a Cas9 nickase with a reverse transcriptase and utilizing a prime editing guide RNA (pegRNA). The pegRNA consists of three parts: a single-guide RNA (sgRNA), a primer binding site (PBS), and a reverse transcription template (RT template) containing the desired edit [17]. The pegRNA guides nCas9 to create a single-strand nick at the target DNA site. The nicked DNA strand binds to the PBS sequence, and the reverse transcriptase synthesizes a new DNA sequence using the RT template, enabling base substitutions, insertions, or deletions. Prime editing can achieve 12 types of base substitutions, small insertions, and deletions, offering a new method to minimize off-target effects and improve target specificity in the genome compared to base editors [37]. However, prime editing systems and their applications still require improvements, such as addressing gRNA-dependent or independent off-target effects, high rates of insertions and deletions mediated by PE3, and the delivery of prime editing systems in adult animals. Further research on animal models is needed.

### 2.4. Gene Delivery Systems

The successful application of gene editing technology relies not only on efficient editing tools but also on effective delivery systems that transport editing components to target cells. The choice of delivery system and vector directly affects the efficiency, specificity, and safety of gene editing. Table 2 summarizes the principles and characteristics of different gene delivery systems and introduces several classic delivery vectors.

#### 2.4.1. Viral Vectors

Viral vectors are among the most commonly used tools for gene editing delivery, including lentivirus (Lentivirus) vectors, adenovirus (Adenovirus) vectors, and adeno-associated virus (AAV) vectors. AAV is widely used for in vivo gene editing due to its low immunogenicity and long-term expression characteristics. Lentiviruses, which can integrate into the host genome, are suitable for in vitro cell editing and stem cell research [45]. However, the biosafety of lentiviral vectors cannot be guaranteed, and AAV vectors can only carry approximately 4.7 kb of foreign DNA, limiting their application due to packaging capacity and potential immune responses [46].

#### 2.4.2. Non-Viral Vectors

Non-viral vectors, due to their low immunogenicity and ease of large-scale production, are increasingly becoming an important choice for gene editing delivery. Lipid nanoparticles (LNPs) are currently the most commonly used non-viral vectors, capable of efficiently delivering CRISPR/Cas9 mRNA and sgRNA. Additionally, polymer nanoparticles such as PEI and PLGA have been extensively studied, with surface modifications further improving targeting and delivery efficiency [47]. Compared to viral vectors, non-viral vectors have lower transfection efficiency and potential cytotoxicity, limiting their in vivo applications.

#### 2.4.3. Nanomaterial Vectors and Cell Delivery

In recent years, novel nanomaterial vectors, such as gold nanoparticles, carbon nanotubes, and metal–organic frameworks (MOFs), have shown great potential in gene editing delivery. These materials offer high loading capacity, good biocompatibility, and controllable release properties. MOFs, due to their degradability and high loading capacity, have been used to deliver CRISPR/Cas9 complexes, significantly improving editing efficiency. Additionally, modifying the surface of vectors with antibodies or ligands enables targeted delivery to specific cell types, enhancing the precision of gene editing. In cancer therapy, nanoparticles have successfully delivered the CRISPR/Cas9 system to tumor cells, significantly improving treatment efficacy and reducing off-target effects [48]. Cell-penetrating peptides (CPPs) have also been used to enhance the intracellular delivery efficiency of gene editing components [49].

The advancement of gene editing technologies has profoundly influenced the iterative innovation of techniques in the livestock sector. The following table presents a timeline of major milestones in the development of gene editing technology across various livestock species (Table 3).

Building on this broader context of livestock applications, the following section focuses specifically on the application of gene editing technology in poultry.

## 3. Applications of Gene Editing Technology in Poultry Animals

**Figure 5:**
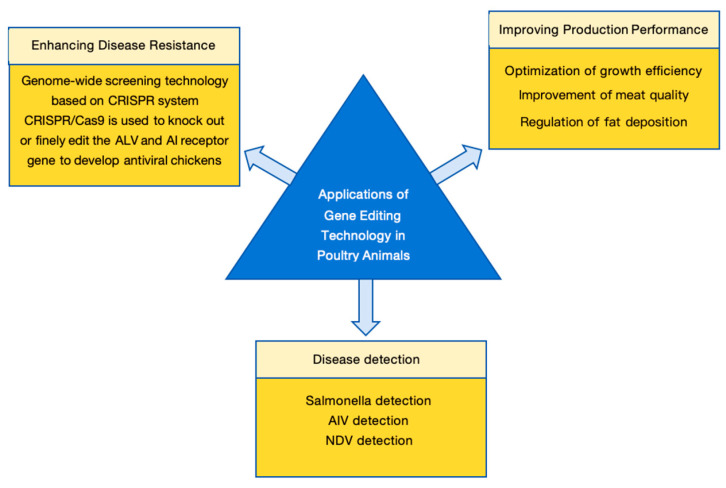
Applications of gene editing in poultry animals. The application of gene editing technology in the poultry industry is primarily categorized into three strategic domains: (1) Enhancing disease resistance by targeting host susceptibility factors or viral receptors (e.g., ALV and AIV resistance); (2) Improving production performance, including the optimization of growth efficiency, modulation of fat deposition, and enhancement of meat quality traits; (3) Facilitating rapid and highly sensitive disease detection utilizing CRISPR-based diagnostic systems (e.g., CRISPR-Cas12a/Cas13a for Salmonella, AIV, and NDV surveillance).

The development of CRISPR/Cas9-mediated genome editing technology is crucial for improving gene targeting efficiency, with the potential to rapidly enhance disease resistance, disease detection, and product quality traits in poultry and other agricultural animals, thereby improving the safety, controllability, and environmental benefits of gene-edited animal breeding (Figure 5). Table 4 summarizes the main applications of gene editing technology in poultry, organized by application area, target genes, methods, and achievements.

### 3.1. Enhancing Disease Resistance

Gene editing technology has shown significant advantages in the research and control of animal viral diseases. Strategies for controlling animal viral diseases include precise modification of the host genome to enhance resistance to viral infections and modification of the viral genome to reduce its replication and transmission capabilities. This technology has been widely applied in poultry for diseases such as Avian Leukosis, Newcastle disease virus (NDV) and Avian Influenza virus (AIV).

#### 3.1.1. Genome-Wide Screening Technology Based on CRISPR System

In recent years, genome-wide CRISPR library screening technology has found widespread utility in diverse fields, including the development of disease-resistant strains, the establishment of disease models, and the design and advancement of vaccines—all achieved by precisely modulating the expression of host factors. The intricate crosstalk between viruses and host cells adds further complexity to disease treatment, rendering the identification of host factors involved in virus–host interactions a critical hurdle for precise therapeutic interventions. Benefiting from the progress of CRISPR gene-editing technology, this screening approach has been successfully applied to pinpoint key antiviral host factors in poultry. Numerous studies have confirmed that CRISPR library screening can effectively identify host factors that engage with viruses across multiple stages of infection, such as adsorption, endocytosis, and replication. These identified host factors offer valuable molecular insights for the prevention and treatment of viral diseases.

During the viral adsorption phase, genome-wide screening has uncovered core regulatory factors including LDLR, SLC35A1, and SLC35B2. These factors either act as viral receptors, participate in receptor biosynthesis, or compete with viruses for receptor binding (e.g., B4GALNT2), thereby governing the initial attachment of viruses to host cells.

In the viral endocytosis stage, screened factors such as SGMS1, IGDCC4, COG8, CYTH2, and PKCθ have been shown to be involved in transmembrane transport and intracellular vesicular trafficking, facilitating viral internalization into host cells and subsequent intracellular transport processes.

At the viral replication stage, factors like TMEM41B, the GARP/EARP complex, KXD1, SMPDL3B, and ZD17 have been discovered. These factors regulate intracellular membrane dynamics, autophagic processes, and lipid metabolism, ultimately influencing viral genome replication and viral particle assembly.

In the context of antiviral immune responses, two categories of key factors have been identified: one comprises antiviral factors (e.g., STAG2 activates the cGAS-STING signaling pathway, while JADE3 promotes the expression of the antiviral protein IFITM3), and the other includes factors that assist viruses in immune evasion (e.g., HSP90AB1 stabilizes viral proteins, and SLA-DM aids African swine fever virus in evading host immune surveillance) [50].

#### 3.1.2. CRISPR/Cas9 Is Used to Knock out or Finely Edit the ALV and AI Receptor Gene to Develop Antiviral Chickens

##### Avian Leukosis

Avian Leukosis (AL) is a contagious tumor disease caused by Avian Leukosis virus (ALV), primarily affecting chickens and other poultry [71]. ALV belongs to the retrovirus family and integrates its genome to the host cell DNA, leading to cell transformation and tumor formation ALV is divided into several subgroups (e.g., A, B, C, D, E, and J) based on envelope protein differences, with subgroup J (ALV-J) being highly pathogenic and having a broad host range, making it the most harmful subgroup to the poultry industry in recent years [72]. Different ALV subgroups utilize different host receptors, such as TVA for subgroup A and the chicken type I Na+/H+ exchanger (chNHE1) for subgroup J. W38 is a key amino acid residue in chNHE1, and its deletion may confer resistance to ALV-J [51,73]. Gene editing technology offers a new strategy for controlling ALV by targeting the viral receptor gene or the viral genome to block ALV infection and replication. The CRISPR/Cas9 system, due to its high efficiency and precision, has become the preferred tool for ALV research. Lee et al. used CRISPR/Cas9 to introduce a W38 mutation in chNHE1 in primordial germ cells (PGCs) and transplanted them into rooster reproductive systems, producing W38-deficient gene-edited chickens that were completely resistant to ALV-J [51]. Koslová et al. introduced deletion mutations in TVA and TVC, creating DF-1 cells resistant to ALV-A and ALV-C, respectively [52]. In addition to editing host genes, CRISPR/Cas9 can directly target the ALV genome to disrupt its replication and transmission. Chen et al. used a replication-competent retroviral vector to deliver shRNA-mirs targeting ALV-B, significantly inhibiting ALV-B replication and spread in chicken cells [74].

##### Avian Influenza

Avian Influenza (AI) is a highly contagious disease caused by the Avian Influenza virus (AIV), which primarily affects poultry and wild birds. AIV belongs to the orthomyxoviridae family and is classified into multiple subtypes (e.g., H5N1, H7N9) based on surface protein hemagglutinin preprotein (HA) and neuraminidase (NA) [75]. Highly pathogenic Avian Influenza viruses (HPAIV) can cause severe respiratory illness and high mortality in poultry, posing a significant threat to global poultry production and public health [76]. AIV enters cells by binding its HA protein to sialic acid receptors on the surface of host cells, releasing RNA genomes, and utilizing host cell mechanisms for replication and transcription. In the process of viral replication, gene rearrangement may occur, leading to the emergence of new subtypes that are more transmissible and pathogenic. Therefore, Jin SePark et al. introduced human β-1,4N-acetylgalactosamine transferase 2 (B4GALNT2) into chicken fibroblasts to modify the sialic acid glycan chain containing α-2,3 ligation by transferring N-acetylgalactosamine to the subterminal galactose of the glycan chain, thereby playing a key role in preventing viruses from binding to the cell surface. Alewo et al. utilized CRISPR/Cas9 to generate homozygous gene-edited (GE) chickens containing two ANP32A amino acid substitutions to prevent viral polymerase interactions. Most vaccination strategies to control AIV have limited effectiveness, so AIV testing remains the main way to control the spread of the disease. Gene editing has been applied to AIV detection. Zhou et al. developed a rapid AIV detection system based on CRISPR/Cas12a, which demonstrates high sensitivity without the need for laboratory equipment [66]. CRISPR/Cas13a-based AIV detection systems have also been reported for H9N2 isoforms with high accuracy.

### 3.2. Improving Production Performance

As the core pillar of the global animal protein supply, the poultry industry has always been a core goal in the field of breeding to improve its production performance (such as optimization of growth efficiency, improvement of meat quality, and regulation of fat deposition). Although traditional breeding methods have achieved certain results in breed improvement, they have limitations such as long cycle, low accuracy, and strong dependence on genetic background, which are difficult to meet the development needs of modern poultry industry “efficient, high-quality and green”. The rise of gene editing technology, especially the CRISPR/Cas9 system, has provided a revolutionary tool for the precise improvement of poultry production performance—through the targeted modification of key functional genes, it can directly regulate core traits such as poultry growth and development, fat metabolism, etc., and break through the genetic bottleneck of traditional breeding.

#### 3.2.1. Optimization of Growth Efficiency

Growth efficiency is the core economic trait of the poultry industry, which mainly depends on the growth rate, feed conversion rate and muscle growth potential. Relevant research focuses on key genes of growth regulation pathways and achieves precise trait improvement through gene editing.

Nawaz et al. systematically analyzed the genetic regulatory network of poultry growth by integrating genomics, transcriptomics, and single-cell RNA sequencing (scRNA-seq) data. The genetic regulatory network of poultry growth was systematically analyzed. The study clarified that myostatin (MSTN) is a key gene that negatively regulates skeletal muscle growth, and its knockout can lift the inhibition of muscle growth, increase the body weight of chickens by 26.9%, while inducing skeletal muscle hypertrophy and reducing abdominal fat deposition, achieving the dual effect of “muscle gain and fat loss”. In addition, key targets such as IGF2BP1, CAB39L, and LCORL were screened—IGF2BP1 by regulating cell proliferation, differentiation, and metabolism, while poultry weight and muscle weight were affected, and the deletion of promoter regions could significantly promote muscle growth. CAB39L is involved in energy balance and nutrient metabolism; editing this gene can optimize the activity of growth-related pathways. LCORL and LDB2 are localized in the growth-related QTL hotspots of chicken chromosomes 1 and 4, improving the size and growth rate of poultry by regulating mitochondrial function and skeletal muscle development. It was also reported that CRISPR/Cas9 technology was used to edit the above genes in chicken primitive germ cells (PGCs), and a transgenic strain with significantly improved growth rate and feed use efficiency was successfully cultivated, providing a complete technical solution for directed improvement of growth efficiency in targeted lines, including foundational studies often performed on commercial broiler backgrounds such as Ross 308 or specific local breeds (e.g., White Feather broilers) [54].

#### 3.2.2. Improvement of Meat Quality

As a positive regulator of adipocyte differentiation, ZNF423 can promote intramuscular fat deposition by activating the expression of lipogenic marker genes such as PPARG, FABP4, and CEBPA, and intramuscular fat content is positively correlated with meat tenderness, juiciness, and flavor. NR2F2 inhibits excess fat accumulation by specifically binding to the distal promoter region of ZNF423 (−2356 to −2346 bp) through its DNA-binding domain (72–143 aa), thereby down-regulating the transcriptional expression of ZNF423. By regulating the expression balance of the two through gene editing technology, the goal of “reducing abdominal fat deposition and retaining an appropriate amount of intramuscular fat” can be achieved, effectively solving the negative correlation between fat content and meat quality in traditional breeding, and taking into account the growth efficiency and meat quality optimization [55].

PLIN1, as the most abundant structural protein on the surface of lipid droplets, maintains the dynamic balance of lipid storage and decomposition by regulating the binding efficiency of lipases (ATGL, HSL) to neutral lipids. CRISPR/Cas9-mediated PLIN1 gene knockout in chicken preadipocytes can significantly improve meat quality: on the one hand, it increases the proportion of unsaturated fatty acids in intramuscular fat, reduces the content of saturated fatty acids, and improves the nutritional value and flavor of meat. On the other hand, it inhibits excessive fat deposition and avoids the loss of tenderness caused by muscle fibers being wrapped in fat. At the same time, it can indirectly regulate the expression of genes related to muscle fiber development, such as MYOD1 and MYOG, optimize the shape and density of muscle fibers, and further improve the tenderness of meat [56].

The regulatory network composed of myogenic factors such as MYOD1, MYOG, and MYF5 directly participates in the determination and density regulation of skeletal muscle fiber type, regulates their spatiotemporal expression mode through gene editing technology, which can increase the proportion of slow-twitch muscle fibers and significantly improve the tenderness and juiciness of meat. Targeted editing of fatty acid metabolism-related genes such as FADS2 and PRKAG3 can further optimize the fatty acid composition of intramuscular fat, increase the content of unsaturated fatty acids, and enhance the nutritional value of meat [54].

#### 3.2.3. Regulation of Fat Deposition

Li et al. systematically elucidated the mechanism of action of the NR2F2-ZNF423 transcriptional regulatory axis in fat deposition. Overexpression of NR2F2 as an inhibitor can significantly inhibit the differentiation of chicken preadipocytes (oil red O staining showed a decrease in lipid droplet formation); Overexpression of ZNF423 activates the fat differentiation pathway and promotes lipid droplet accumulation. Supplementation with ZNF423 in NR2F2-overexpressing cells can reverse the phenotype of fat deposition inhibition, confirming that the upstream and downstream regulatory relationship between the two is the core switch of fat deposition. Transcriptome analysis showed that PPAR signaling pathway and fatty acid synthesis pathway were significantly enriched in ZNF423 overexpressing cells, providing a pathway-level reference for multi-target co-editing [55].

Zhai et al. targeted the functional validation of the PLIN1 gene, revealing a novel pathway for the regulation of lipid droplet metabolism. PLIN1 balances basal lipolysis with hormone-stimulated lipolysis processes by regulating the activity of key lipolytic enzymes (ATGL, HSL). After CLISPR/Cas9 knockout of PLIN1, chicken preadipocytes showed the characteristics of “enhanced basal lipolysis and hormone-stimulated lipolysis inhibition”: the content of triglycerides (TG) was significantly reduced in the basal state, and the activities of ATGL and HSL were increased. After hormone stimulation, on the contrary, it effectively reduces the excessive accumulation of abdominal fat. At the same time, PLIN1 knockout cell morphology changed from spindle to elliptical, increasing proliferation and reducing early apoptotic activity, further inhibiting adipose tissue hyperproliferation and providing a complete cellular mechanism for the regulation of fat deposition [56].

Nawaz et al. found that growth signaling pathway genes (IGF2BP1, CAB39L) can indirectly affect fat distribution by regulating energy metabolism and nutrient absorption, reducing fat deposition to the abdomen, avoiding the negative correlation between growth efficiency and excessive fat deposition, and providing a multi-target option for “growth-fat” synergistic optimization [54].

### 3.3. Disease Detection

#### 3.3.1. *Salmonella* Detection

*Salmonella* (especially *Salmonella typhimurium*) is one of the main pathogens causing contamination of poultry products, with high contamination rates and widespread routes, and rapid testing is critical to food safety. Jia et al. developed a CRISPR-SERS biosensor without amplification steps. The sensor targets the invasin A gene (invA) of *Salmonella typhimurium*, activates trans-cleavage activity after recognizing the target sequence via the Cas12a protein, cleaves the Rox-labeled single-stranded DNA reporter probe fixed on a silver nanoparticle-modified anodized aluminum (AAO) substrate, and then uses surface-enhanced Raman spectroscopy (SERS) to detect signal changes. The platform eliminates the need for DNA amplification steps, has a detection limit as low as 110 CFU/mL, has a recovery rate of 87.4–115.0% in chicken samples, and can specifically distinguish *Salmonella* from other pathogens such as *Escherichia coli* and *Vibrio parahaemolyticus*, and the entire detection process can be completed within 2 h, providing an efficient solution for on-site detection of *Salmonella* in the poultry production chain [57].

#### 3.3.2. AIV Detection

Avian Influenza virus (AIV) is one of the most harmful poultry viruses with multiple subtypes and rapid mutations, and rapid detection is crucial for epidemic prevention and control. Xu et al. developed a CRISPR-Cas13a-mediated fluorescence detection platform to design specific crRNAs for the hemagglutinin gene (HA) of the Avian Influenza virus H9 subtype, and when viral RNA is present, Cas13a is activated and cleaves the fluorescently labeled RNA reporter probe, releasing a fluorescent signal. The method has a detection limit of 10 copies/μL, a detection time of only 1 h, and can distinguish between H9 subtypes and other Avian Influenza subtypes, making it suitable for rapid on-site screening [77].

#### 3.3.3. NDV Detection

Newcastle disease virus (NDV) is an important pathogen that causes devastating diseases in poultry, Li et al. constructed an electrochemical sensor based on CRISPR-Cas12a, which targets the fusion protein gene (F gene) of Newcastle disease virus, and activates trans-cleavage activity after recognizing the target DNA through Cas12a, cutting the nucleic acid probe on the electrode surface, resulting in changes in electrochemical signals. The sensor has a detection limit of up to 5 copies/μL and can effectively resist matrix interference in poultry tissue samples, providing a reliable solution for the early diagnosis of Newcastle disease virus [78].

### 3.4. Applications in Commercial Poultry Lines vs. Purebred Lines

A critical consideration in the deployment of gene editing technology is its applicability to commercial poultry lines versus purebred lines. Currently, the vast majority of foundational gene-editing research and successful proof-of-concept studies in poultry are conducted using purebred experimental lines (such as White Leghorn layer lines) or specific laboratory cell lines (such as DF-1). The primary reason is that purebred lines possess well-characterized, stable, and homogenous genetic backgrounds, which facilitate the precise evaluation of the edited gene’s phenotypic effects without the confounding variables of hybrid genetics.

However, modern commercial poultry production relies entirely on highly complex, multi-way crossbred hybrid lines (e.g., commercial broilers like Ross 308 or Cobb 500) to maximize heterosis (hybrid vigor). Direct genetic editing of these commercial terminal hybrids is both technically impractical and biologically inefficient due to their complex genetic segregation and the inability to pass these traits reliably to the next generation (as commercial hybrids are not used for breeding).

Therefore, the successful integration of gene editing into commercial poultry requires a top-down introgression strategy within the commercial breeding pyramid. The target gene edits (e.g., the W38 mutation for ALV-J resistance or ANP32A edits for AIV resistance) must first be generated and established as homozygous traits in the purebred great-grandparent (GGP) or pedigree lines at the apex of the breeding pyramid. Once the desired edit is fixed in these purebred elite lines, the trait can be naturally cascaded down through the grandparent (GP) and parent stock (PS) generations via conventional crossbreeding. This strategy ensures that the millions of commercial birds at the base of the pyramid ultimately inherit the beneficial gene-edited traits (such as enhanced disease resistance) while fully preserving the finely tuned production traits and heterosis of the commercial hybrid lines. Consequently, while the editing process itself is strictly performed on purebred lines, its ultimate impact and economic value are profoundly realized in commercial poultry lines.

## 4. Risks and Challenges of Gene Editing Technology

Despite significant progress in the application of gene editing technology in agricultural animals, several challenges remain. Off-target effects compromise the biosafety of gene-edited animal products, ethical controversies and social acceptance limit the further market of gene-edited agricultural animals, and the technical complexity demands high expertise from researchers. Developing more precise gene editing tools, stricter off-target detection methods, establishing unified international regulatory frameworks, and creating more efficient delivery systems and simpler operational methods may address these issues.

### 4.1. Off-Target Effects and Safety

Off-target effects refer to accidental mutations introduced by gene editing tools at non-target sites, which can lead to cellular dysfunction and even cancer, which is a major risk of gene editing technology [79]. Although CRISPR/Cas9 systems offer higher targeting specificity compared to earlier technologies, there are still non-targeting risks in complex genomic environments [80]. Studies have shown that gRNA can bind to sequences with 3–5 base mismatches on the target DNA, causing Cas9 to cleavage at unintended sites, which in turn leads to genomic instability [81].

To reduce off-target effects, the researchers have developed several strategies. For example, high-fidelity Cas9 variants such as eSpCas9 and SpCas9-HF1 optimize Cas9 protein structure and significantly reduce the off-target rate [82]. The double scratch strategy has also been shown to reduce the rate of deviation from the target by 50 to 1500 times [83,84]. In addition, the use of Cas9 Nickase, a mutant that introduces only single-strand breaks rather than double-stranded breaks, can effectively enhance the specificity of editing, which significantly reduces off-target effects compared to traditional Cas9 by requiring two gRNAs to target opposite strands near the target editing site [85].

However, these methods cannot completely eliminate off-target effects. The long-term effects of non-target effects are unclear, especially in the germline and embryo editing areas. Therefore, the development of more accurate gene editing tools and more rigorous off-target detection methods are key directions for future research [86].

### 4.2. Ethical and Legal Issues

The rapid development of gene editing technology has sparked widespread ethical and legal debates, particularly in the field of human germline and embryo editing. In 2018, He Jiankui’s team announced the successful editing of the CCR5 gene in human embryos, triggering global ethical controversy [87]. Tomita et al. also pointed out that DSB-based gene editing in human embryos may cause chromosomal breaks, large fragment deletions, or rearrangements, and their safety has not been fully verified [88].

This event underscored the complexity of gene editing technology in terms of ethics and law, particularly in areas involving human life origins and genetic modification. The core of the ethical debate lies in the potential misuse and irreversibility of gene editing technology. Since there is no guarantee that gene-edited humans will not produce offspring, the consequences of germline editing may be inherited by future generations, with profound impacts on human society [89]. Additionally, the commercialization of gene editing technology may lead to the emergence of “designer babies,” exacerbating social inequality [90]. In the field of agricultural animals, even if off-target mutations are not introduced, gene editing may have unintended effects on the growth and reproductive performance of individuals.

Therefore, many countries and international organizations have called for strict ethical guidelines and legal frameworks to regulate the application of gene editing technology [91]. Legally, the regulation of gene editing technology is still in its exploratory stages. The European Union and the United States have significantly different regulatory policies for gene-edited crops, with the EU treating them as genetically modified organisms (GMOs) [92]. These policy differences not only limit the application of gene editing technology in animal husbandry but may also lead to international trade disputes. Therefore, establishing a unified international regulatory framework is an important future task [93].

### 4.3. Technical Complexity

The operational complexity of gene editing technology is one of the main obstacles to its widespread application. First, the design and optimization of gene editing tools require a high level of expertise and experience. For example, the design of sgRNAs must consider factors such as target sequence specificity, GC content, and secondary structure to ensure editing efficiency and accuracy [94].

Efficiently delivering gene editing components to target cells or tissues is also a core technical challenge. Different delivery methods have their respective advantages and limitations, and no universal optimal solution exists. For example, adeno-associated virus (AAV) vectors are limited by packaging capacity, leading researchers to develop dual AAV split strategies or use smaller Cas9 homologs such as SaCas9 and Cas12f [95]. Novel delivery platforms, such as bacterial extracellular contractile injection systems (eCIS), can specifically recognize target cells and deliver large molecules (e.g., SpCas9 protein and zinc-finger deaminases) with high specificity and low immunogenicity [96], providing new approaches for gene editing delivery.

DNA double-strand breaks (DSBs) are primarily repaired via non-homologous end joining (NHEJ) or homology-directed repair (HDR). NHEJ occurs efficiently in all cell cycle stages but tends to introduce random insertions or deletions (indels), whereas HDR requires a donor DNA template and is mostly limited to the S/G2 phase, with lower efficiency than NHEJ [81,95]. For scenarios requiring precise knock-in or point mutation repair, HDR’s low efficiency is a major bottleneck. Recently developed base editors (BEs) and prime editors (PEs) allow single-base conversions or small insertions/deletions without generating DSBs, bypassing HDR limitations, though they still have constraints such as deaminase-mediated off-target effects and limited editing windows. Notably, the NICER technique introduces multiple single-strand nicks near heterozygous mutation sites using nCas9, leveraging homologous chromosomes as endogenous repair templates for precise correction, with minimal DSB-related unintended mutations [88].

## 5. Conclusions

Gene editing technologies, particularly CRISPR/Cas9 systems, have shown significant potential in agricultural animal research. By precisely editing genomes, disease resistance, productivity, and disease detection can be rapidly improved. It has shown significant accuracy and efficiency in improving disease resistance (e.g., ALV and AIV) [97]. In studies aimed at improving animal performance, editing the MSTN gene reduced the deposition of avian fat in the abdomen to improve muscle mass and increase palatability [55]. These achievements not only improve the economic efficiency of livestock farming but also provide new solutions to global food security challenges. Although significant advances have been made in the application of gene editing technology in agricultural animals, it still faces several challenges, including off-target effects, ethical controversies, technical complexity, and social acceptance. Off-target effects are a major challenge of gene editing techniques. Reducing non-target effects requires more precise gene editing tools such as Cas9 variants, as well as the development of base and prime editors. In addition, more efficient delivery systems are needed to improve the efficiency and specificity of gene editing. Novel nanomaterial carriers, such as gold nanoparticles and metal–organic frameworks, have high loading capacity, good biocompatibility, and controlled release properties, which are key factors driving the application of CRISPR/Cas9 systems [98]. Cell-penetrating peptides (CPPs) enter cells through electrostatic interactions or endocytosis, making them suitable for delivering various macromolecules and cells, with low immunogenicity and toxicity. CPP-based delivery systems have great potential for in vivo applications [99]. The development of gene editing technology relies on interdisciplinary research. For example, integrated genomics, proteomics, and metabolomics can comprehensively assess the impact and potential risks of gene editing. Combining artificial intelligence and big data technology to optimize the design and delivery strategies of gene editing tools can improve editing efficiency and accuracy [100]. The ethical and legal controversies surrounding gene editing technology are also major obstacles to its widespread adoption. Genetic modification introduced into animals can have permanent deleterious effects on species unless genetic programs are used continuously to prevent deleterious mutations from entering the gene pool. The commercialization of gene editing technology may affect existing animal welfare. For example, some traits produced by gene editing can occur naturally in populations, while profit-driven genetic modification may ignore existing animal welfare issues [101]. Establishing a unified international regulatory framework to ensure the safety and fairness of gene editing technology can prevent unnecessary suffering in animals. In addition, strengthening public education and participation in gene editing technology is an important way to increase social acceptance. Gene editing technology has made significant progress in poultry breeding, providing new solutions to improve disease resistance, production performance, animal welfare, and reduce environmental pollution. However, gene editing technology still faces many challenges, including target bias, ethical controversies, technical complexity, and social acceptance. In the future, with the continuous advancement and optimization of technology, gene editing technology is expected to achieve wider applications in the field of agricultural animals and make important contributions to global food security and sustainable development.

## Figures and Tables

**Table 1:** Development of Gene-Editing Technologies and Their Applications in Poultry.

Time	Technology	References
1970	Restriction enzymes	A restriction enzyme from *Hemophilus influenzae*. I. Purification and general properties [5]
1972	DNA recombination in vitro	Biochemical method for inserting new genetic information into DNA of Simian Virus 40: circular SV40 DNA molecules containing lambda phage genes and the galactose operon of *Escherichia coli* [6]
1994	Homologous recombination	Targeted gene replacement [7]
1997	Cloning of the sheep	Viable offspring derived from fetal and adult mammalian cells [8]
2001	ZFNs	Stimulation of homologous recombination through targeted cleavage by chimeric nucleases [9]
2011	TALEN	A TALE nuclease architecture for efficient gene editing [10]
2012	CRISPR/Cas9	A programmable dual-RNA-guided DNA endonuclease in adaptive bacterial immunity [1]
2013	CRISPR/Cas9 in eukaryotic cells	Successful application of CRISPR/Cas9 in mammalian cells accelerated its use in biomedical and agricultural research [11]
2016	CRISPR-mediated editing in chicken primordial germ cells (PGCs)	Efficient gene editing in chicken PGCs enabled stable germline transmission and opened a new era for avian transgenic research [12]
2016	Generation of gene-edited chickens	CRISPR/Cas9 technology was successfully used to generate genetically modified chickens, demonstrating its feasibility in poultry breeding [13]
2016	Base editing	Programmable editing of a target base in genomic DNA without double-stranded DNA cleavage [14]
2017	CRISPR/Cas13a	Nucleic acid detection with CRISPR-Cas13a/C2c2 [15]
2018	CRISPR/Cas12	Plant Gene Editing Using FnCpf1 and LbCpf1 Nucleases at Redefined and Altered PAM Sites [16]
2019	Prime editing	Search-and-replace gene editing without double-strand breaks or donor DNA [17]
2020	Antiviral gene editing in poultry	Genome editing targeting host antiviral genes emerged as a promising strategy for improving poultry disease resistance [18]
2020	ALV-J-resistant chickens via NHE1 editing	Precise editing of the chicken NHE1 receptor conferred resistance to ALV-J infection, demonstrating the practical potential of CRISPR-assisted antiviral breeding in poultry [19]
2021	Clinical translation of CRISPR/Cas9	CRISPR/Cas9 therapies entered clinical trials for human genetic diseases, highlighting the translational potential of gene editing [20]

**Table 2:** Principles and characteristics of different gene delivery systems.

Delivery System	Method	Delivery Principle	Characteristics
Chemical delivery	Calcium phosphate [37]	Calcium phosphate-DNA complexes adsorb to the cell membrane and are endocytosed	Not suitable for primary cells, simple to operate but with poor reproducibility
	Cationic liposome [38]	Positively charged liposomes form complexes with negatively charged nucleic acids and are endocytosed	Widely applicable, high transfection efficiency, good reproducibility
	DEAE-dextran [39]	Positively charged DEAE-dextran interacts with the negatively charged phosphate backbone of nucleic acids to form complexes that are endocytosed	Relatively simple, but has some toxic side effects on cells
Biological delivery	Lentivirus [40]	Infects host cells to integrate foreign genes into the chromosome	Suitable for hard-to-transfect cells, primary cells, and in vivo cells
	Adenovirus [41]		Suitable for hard-to-transfect cells, safety considerations required
Physical transduction	Particle bombardment [42]	DNA is precipitated onto microscopic heavy metal particles and delivered via Biolistic particle delivery, with DNA gradually released and expressed in cells	Suitable for epidermal cells, fibroblasts, lymphocyte lines, and primary cells
	Electroporation [43]	High-voltage pulses disrupt the cell membrane potential, allowing DNA to enter through small pores	High cell mortality, large amounts of DNA and cells required
	Microinjection [44]	DNA is directly injected into the nucleus of target cells using micromanipulation	Limited number of transfected cells, primarily used for engineering or transgenic animal embryonic cells

**Table 3:** A timeline of major milestones in gene editing technology for livestock.

Year	Species	Achievement
1997	Sheep	First cloned mammal (Dolly), foundation for gene editing [8]
2015	Pig	MSTN knockout producing double-muscle phenotype [50]
2016	Cattle	POLLED gene editing eliminating need for dehorning [51]
2016	Chicken	First demonstration of gene editing in chicken primordial germ cells [12]
2017	Chicken	Foundational technology enabling rapid detection of viral pathogens in poultry [15]
2017	Chicken	W38-deficient chickens resistant to ALV-J infection [52]
2017	Pig	CD163 knockout conferring complete PRRSV resistance [53]
2018	Goat	BLG knockout reducing milk allergenicity [54]
2018	Chicken	Foundational technology for Cas12a-based detection platforms in poultry [55]
2018	Chicken	DF-1 cells resistant to ALV-A/ALV-C subgroup infection [56]
2021	Cattle	BLG-free cows with reduced milk allergenicity [57]
2022	Pig	KISS1 knockout boars eliminating castration [58]
2023	Chicken	Homology-directed genome editing confers resistance to avian influenza infection [59]
2023	Chicken	Enables rapid, sensitive on-site detection of AIV [60]
2024	Cattle	SLICK1 mutation for heat-stress resistance [61]
2024	Goat	ISDra2-TnpB system for mastitis resistance (no foreign gene) [62]
2024	Chicken	Identification of Cables1 as a critical host factor that promotes ALV-J replication [63]
2025	Chicken	Integrative multi-omics analysis deciphers regulatory mechanisms of production traits [64]

**Table 4:** Applications of Gene-Editing Technology in Poultry: Targets, Genes, Methods, and Achievements.

Application Area		Target	Representative Genes	Key Methods	Achievements
Enhancing disease resistance	Genome-wide antiviral factor screening	Identify host factors at adsorption, endocytosis, replication, or immune evasion	LDLR, SLC35A1, B4GALNT2, SGMS1, TMEM41B, STAG2, HSP90AB1, etc.	CRISPR library-based genome-wide screening	Identifies key host factors in viral lifecycle and antiviral response [65]
	CRISPR/Cas9 editing of ALV and AIV receptors for antiviral chickens	Block ALV-J infection	chNHE1 (W38 residue)	CRISPR/Cas9 knockout or precise editing in PGCs	Produces chickens resistant to ALV-J [52]
		Block ALV-A/ALV-C infection	TVA, TVC	CRISPR/Cas9 knockout in PGCs	DF-1 cells resistant to ALV-A and ALV-C [56]
		Block AIV binding	B4GALNT2, ANP32A	CRISPR/Cas9 knockout or precise editing in PGCs	Modifies sialic acid receptors, prevents viral polymerase interaction [66]
Improving Production Performance	Improving growth efficiency	Increase growth rate, muscle mass	MSTN, IGF2BP1, CAB39L, LCORL, LDB2	CRISPR/Cas9 knockout or promoter editing in PGCs	Enhances skeletal muscle growth, feed conversion, reduces abdominal fat [67]
	Improving meat quality	Optimize intramuscular fat, tenderness, juiciness, muscle fiber type, fatty acid composition	ZNF423, NR2F2, PLIN1, MYOD1, MYOG, MYF5, FADS2, PRKAG3	CRISPR/Cas9-mediated gene editing	Balances fat deposition, enhances meat quality [68,69]
	Regulating fat deposition	Reduce abdominal fat, regulate adipocyte differentiation	NR2F2-ZNF423 axis, PLIN1	CRISPR/Cas9 knockout or overexpression	Controls lipolysis, fat accumulation [68,69]
Disease detection		Rapid detection of Salmonella Typhimurium	InvA	CRISPR-SERS biosensor	Amplification-free, high sensitivity (110 CFU/mL) [70]
Commercial line integration		Introduce disease resistance traits to commercial hybrids	W38 mutation (ALV-J), ANP32A edits (AIV)	Establish edits in homozygous GGP/pedigree lines, propagate through GP/PS	Commercial hybrids inherit traits [19,66]

## Data Availability

No new data were created or analyzed in this study.

## Abbreviations

The following abbreviations are used in this manuscript:

ZFN	Zinc Finger Nucleases
TALEN	Transcription Activator-Like Effector Nucleases
CRISPR	Clustered Regularly Interspaced Short Palindromic Repeats
Cas9	CRISPR-associated protein 9
DSBs	Double-Strand Breaks
NHEJ	Non-Homologous End Joining
HDR	Homology-Directed Repair
nCas9	Nickase Cas9
dCas9	Dead Cas9
PAM	Protospacer Adjacent Motif
crRNA	CRISPR RNA
tracrRNA	Trans-activating
gRNA	Guide RNA
ssODN	Single-Stranded Oligonucleotide
CBEs	Cytosine Base Editors
ABEs	Adenine Base Editors
GBEs	Guanine Base Editors
PEs	Prime Editors
pegRNA	Prime Editing Guide RNA
LNP	Lipid Nanoparticles
PEI	Polyethylenimine
PLGA	Poly (lactic-co-glycolic acid)
MOFs	Metal–Organic Frameworks
CPPs	Cell-Penetrating Peptides
PRRSV	Porcine Reproductive and Respiratory Syndrome Virus
CD163	Cluster of Differentiation 163
PRNP	Prion Protein Gene
MSTN	Myostatin
BLG	Beta-lactoglobulin
FGF5	Fibroblast Growth Factor 5
ATGL	Adipose Triglyceride Lipase
G0S2	G0/G1 Switch Gene 2
CH4	Methane
TB	Tuberculosis
NRAMP1	Natural Resistance-Associated Macrophage Protein 1
SP110	Sp110 Nuclear Body Protein
LEPR	Leptin Receptor
KISS1	Kisspeptin1
ALV	Avian Leukosis Virus
AIV	Avian Influenza Virus
HA	Hemagglutinin
NA	Neuraminidase
HPAIV	Highly Pathogenic Avian Influenza Virus
RNA	Ribonucleic Acid
DNA	Deoxyribonucleic Acid
mRNA	Messenger RNA
sgRNA	Single Guide RNA
shRNA	Short Hairpin RNA
miRNA	MicroRNA
siRNA	Small Interfering RNA
CRISPRa	CRISPR Activation
AAV	Adeno-Associated Virus
ALV-J	Avian Leukosis Virus Subgroup J
NDV	Newcastle Disease Virus
PGC	Primordial Germ Cell
SCNT	Somatic Cell Nuclear Transfer
SERS	Surface-Enhanced Raman Scattering
Rox	6-Carboxy-X-Rhodamine
PS	Phosphate-Buffered Saline
GGP	Grandparent Generation Poultry
GP	Germline Progenito
